# The locus coeruleus at the crossroads of inflammation and neurodegeneration in multiple sclerosis

**DOI:** 10.1186/s12974-026-03981-2

**Published:** 2026-08-26

**Authors:** Intakhar Ahmad, Rebecca Alemani, Douglas L. Feinstein

**Affiliations:** 1 Department of Anesthesiology, College of Medicine, University of Illinois Chicago, 835 S Wolcott Ave, Chicago, IL 60612 USA; 2 Department of Veterans Affairs, Jesse Brown VA Medical Center, Chicago, IL USA

**Keywords:** Multiple sclerosis, Locus coeruleus, Noradrenaline, Myelin, Toxic metals

## Abstract

**Background:**

Multiple sclerosis (MS) is increasingly recognized as a systems-level disorder encompassing grey matter injury, network disconnection, and neuropsychiatric symptoms alongside demyelination. Within this framework, the locus coeruleus (LC), the principal source of noradrenaline (NA) in the central nervous system, emerges as a plausible regulatory hub within a distributed monoaminergic network.

**Main body:**

LC-derived NA shapes microglial and astrocytic reactivity, oxidative stress, mitochondrial function, oligodendrocyte differentiation, and blood-brain barrier stability through adrenergic receptor signaling. Neuropathological studies show LC neuron loss, astrogliosis, and altered NA levels in MS and related animal models. Post-mortem findings suggest that neuromelanin-bound metals and redox-active trace elements selectively accumulate in LC neurons, potentially triggering mitochondrial dysfunction, barrier disruption, and chronic glial activation. In animal models, pharmacological or genetic reductions in NA worsen inflammation, demyelination, and axonal damage, while increasing NA improves disease outcomes and restores glial and cytokine profiles. Early clinical studies of β_2_-adrenergic agonists and NA-modulating drugs suggest benefits across specific symptom domains, including relapse activity, fatigue, mood, bladder function, and selected cognitive measures, but remain limited by small cohorts and insufficient stratification of patients.

**Conclusions:**

Together, these findings support LC-NA dysfunction as a targetable aspect of MS pathobiology and justify biomarker-driven trials that combine noradrenergic interventions with molecular indicators of glial activity, myelin integrity, and stress-related signaling.

## Introduction

Multiple sclerosis (MS) is increasingly seen as a disorder affecting distributed central nervous system (CNS) networks rather than just a focal white-matter (WM) demyelinating disease. Cortical and deep grey matter (GM) lesions, diffuse neurite injury, synapse loss, and GM atrophy are strongly linked to disability, depression, fatigue, and cognitive impairment. In this broader view, neuromodulatory brainstem nuclei, especially the locus coeruleus (LC), have gained attention as potential contributors to MS-related network dysfunction. The LC provides most of the brain’s noradrenergic (NAergic) input and regulates arousal, attention, stress responses, and mood. Beyond classical neurotransmission, noradrenaline (NA) restrains glial inflammatory activation, supports neurotrophic signaling, and limits oxidative stress through β-adrenergic receptor pathways [1–3]. Loss of LC neurons and reduced central NA levels are well characterized in Alzheimer’s disease (AD) and Parkinson’s disease (PD) [4, 5]; animal studies in these diseases show that experimental LC lesion worsens pathology, whereas restoring NA levels confers benefit [6, 7]. Comparable LC abnormalities with reduced NA are increasingly documented in MS [2, 8].

In MS and experimental autoimmune encephalomyelitis (EAE), pathology in the LC and decreased NA levels were observed alongside increased glial activation, and showed only a weak association with focal plaque burden [8]. Structural and functional imaging studies also show disruption of monoaminergic pathways and changes in network connectivity, linking the LC and other brainstem nuclei to fatigue, slowed cognition, and selective attention deficits [9–12]. Furthermore, the LC’s proximity to blood vessels and its high neuromelanin content have led to the hypothesis that it may serve as an entry point for environmental toxic metals capable of triggering or worsening MS and other CNS disorders [13, 14]. The exact causes of LC damage and NA depletion in MS are not fully understood and are likely to be multifactorial. Plausible contributors include loss of trophic support from widespread LC projection fields, disruption of the blood-brain barrier (BBB) and neurovascular unit, which may increase LC exposure to circulating inflammatory mediators and toxins, and intrinsic vulnerability conferred by dense vascularization, high metabolic demand, and the metal-binding capacity of neuromelanin-containing neurons.

While the importance of the LC-NA system in AD and PD has been documented for over 50 years [4, 5], its role in the progression of MS has been comparatively less studied. To better understand how the LC-NA system influences MS, we review four related topics: (i) the biology and anti-inflammatory functions of the LC-NA system; (ii) evidence for LC and broader monoaminergic network involvement in MS; (iii) mechanisms that may underlie LC vulnerability, including toxicants and barrier dysfunction; and (iv) preclinical and clinical studies targeting the LC-NA axis. Finally, we propose a systems-level model linking brainstem vulnerability to neuroinflammation, demyelination, neuropsychiatric symptoms, and therapeutic opportunity in MS.

## Biology of the LC-NA System

### Anatomy, projections, and projection topography

Several reviews summarize the history of LC anatomy, its projections, and its functions [2, 15–20]. In brief, the LC is a compact pontine nucleus located next to the fourth ventricle that contains tyrosine hydroxylase (TH)-positive NAergic neurons projecting throughout the brain and spinal cord. The LC was first described in the late eighteenth century by Félix Vicq d’Azyr [21] and was later described in the early nineteenth century in the human brain by Johann Christian Reil [22]. The area was named locus coeruleus in 1812 by Joseph and Karl Wenzel [23], based on its blue appearance; at the beginning of the twentieth century, it was termed the “nucleus pigmentosus pontis” because of the presence of melanin granules [24]. Early studies that relied on counting pigmented cells reported between 9,500 and 16,900 cells per LC side [25–27]. Subsequent staining for TH and dopamine β-hydroxylase (DBH) revealed between 45,000 and 60,000 total LC neurons in healthy adult humans [28, 29], with approximately 1,500 in adult mice [30, 31] and 3,600 in adult rats [32, 33].

LC projections innervate cortical association areas, thalamus, cerebellum, limbic structures, brainstem autonomic nuclei, and the dorsal horn of the spinal cord. LC neurons also exhibit chemical heterogeneity. Quantitative analyses and three-dimensional reconstruction studies indicate that the LC is topographically organized, with partially segregated neuronal populations projecting to cortical, cerebellar, autonomic, and spinal targets [26, 28]. Furthermore, in addition to classical NAergic transmission, LC neurons co-release neuropeptides, notably galanin, and potentially other modulators such as neuropeptide Y, enkephalins, and somatostatin within specific cellular subpopulations or circuit contexts [34, 35]. These co-transmitters can influence stress responsiveness, inflammatory tone, and neuronal survival over timescales that differ from that of NA itself [36].

Although the LC is often treated as a homogeneous nucleus, genetic, molecular, anatomical, and electrophysiological studies show that it is organized into partially segregated modules [37, 38]. Distinct, largely non-overlapping LC neurons preferentially innervate specific targets and possess distinct intrinsic properties, allowing targeted rather than purely global neuromodulation [37, 38]. Projection-specific manipulations dissociate functions previously attributed to the LC as a whole; for example, spinal-projecting LC neurons mediate descending analgesia, whereas prefrontal-projecting neurons mediate aversion and anxiety-like behavior [37]. Specific neuronal subpopulations in the LC have been identified by a variety of techniques. These include viral tracing [39], electrophysiology, optogenetics and in situ hybridization [40], single-cell [35, 41] and single-nuclear [42] RNA sequencing, as well as functional connectivity studies using fMRI in humans [43]. These methods have shown heterogeneity in LC neuropeptide and receptor expression, electrical waveform responses, age-related decreases in rostral LC connectivity [44], and selective responses to alpha2-adrenergic receptor activation [43]. Recent molecular analyses using single-cell (sc) or single-nucleus RNA sequencing (snRNAseq) provide evidence for LC neuronal subpopulations in both human [42] and mouse [35, 41]. These findings have not only defined specific neuronal subtypes, but have also revealed heterogeneity of co-expressed neurotransmitters and their receptors within the same neurons, for example co-expression of cholinergic markers [42], and lack of co-expression of NPY but co-expression of galanin [35] in NAergic populations. Importantly, studies suggest that LC heterogeneity can influence neuronal vulnerability. As examples, tau accumulation in AD brains is greater in areas of the dorsal LC that preferentially project to AD-vulnerable regions such as HC and neocortex [45], and LC damage occurring in PD patients assessed by MRI shows a heterogeneous pattern of damage [46]. Altogether, the modularity within the LC may be directly relevant to MS since selective injury to specific LC subpopulations or projection modules could produce dissociable deficits in attention, mood, autonomic control, pain modulation, and anti-inflammatory regulation, helping to explain the clinical heterogeneity of LC-related symptoms.

### Anti-inflammatory, neuroprotective, and myelinogenic properties of NA

A large body of literature demonstrates that activation of β-adrenergic receptors by NA increases intracellular cyclic adenosine monophosphate (cAMP), leading to protein kinase A (PKA)-mediated phosphorylation and activation of cAMP response element-binding protein (CREB), which initiates gene expression changes that suppress pro-inflammatory transcriptional programs [1, 3, 47]. Both in vitro and in vivo studies demonstrate that NA suppresses microglial and astrocytic production of pro-inflammatory cytokines, reduces inducible nitric oxide synthase expression, and protects neurons from excitotoxic injury. This has established the concept of the LC-NA system as an intrinsic anti-inflammatory circuit in the brain, in which endogenous NAergic tone acts as a central brake on neuroinflammatory responses; disruption of LC output is therefore be expected to remove this restraint and facilitate exaggerated glial activation and oxidative stress. NAergic activation of the cAMP/PKA/CREB pathway also engages neurotrophic signaling, promoting expression of brain-derived neurotrophic factor (BDNF) and nerve growth factor (NGF), which support neuronal survival and limit amyloid and excitotoxic injury [48, 49]. In addition, NA regulates astrocytic glutamate buffering that can reduce excitotoxic vulnerability in LC projection regions [50, 51].

NA also has direct actions upon oligodendrocytes (OLGs) and OPCs. Early studies showed that NA stimulates phosphatidyl inositol hydrolysis in OLGs via activation of alpha1-adrenergic receptors (α1-ARs) [52]; that NA and directly beta-adrenergic receptor (β-AR) stimulation using isoproterenol reduced OPC proliferation but stimulated lineage progression [53]. More recent direct optogenetic studies demonstrate that LC-derived NA, acting primarily through α1 and β-ARs, drives arousal-locked calcium transients in cortical OPCs and directly stimulates their proliferation [54, 55] implicating NA as a direct modulator of OPC calcium dynamics in vivo, with implications for remyelination capacity in demyelinating disease [54, 56, 57]. In contrast, it was reported that direct incubation of OLGs with NA induced toxicity [58, 59] involving free radical production; however, that was reduced when co-cultured with astrocytes. Overall, these findings point to effects of NA on OPC proliferation; however, whether NA subsequently biases surviving OPCs toward terminal differentiation and myelination is less certain. Given the multiple consequences of reduced LC-NA output, spanning immune, glial, metabolic, vascular, and synaptic domains, the loss of β-AR restraint on glial cell activation, coupled with resultant declines in cAMP/PKA/CREB-dependent anti-inflammatory and neurotrophic transcription emerges as a likely driver of MS progression, as it simultaneously unleashes neuroinflammation and curtails OLG supportive signaling [60–62].

## Locus coeruleus damage and monoaminergic network disconnection in MS

### Preclinical biochemical evidence of LC-NA dysfunction

Substantial biochemical evidence indicates that the LC-NA system dysfunction develops dynamically during the course of EAE. Early studies reported that NA levels in cerebrospinal fluid and white matter were elevated before clinical signs appeared, but were reduced at later disease stages; NA levels were also lower in the brainstem and spinal cord of EAE rats compared to controls [63–65]. These early compensatory changes appear to be overtaken by progressive LC injury and impairment of the NA synthetic machinery, including TH and dopamine β-hydroxylase. We and others have similarly shown reduced NA levels in the spinal cord and frontal cortex of EAE mice, associated with atrophy and astrocyte activation of TH-positive LC neurons; these alterations occurred in the context of variable local demyelination and inflammatory infiltrates but did not scale directly with plaque burden, suggesting that LC dysfunction is not entirely explained by traditional lesion pathology [8].

In EAE, interventions targeting the LC modify the disease course. Treatment with vindeburnol, a vincamine derivative that enhances TH expression, reduces astrocyte activation, and supports LC neuron survival, improved clinical scores, reduced cerebellar demyelination, and increased spinal cord NA when administered after disease onset [7]. The NA-augmenting agent catalpol increased LC NA output, elevated spinal cord NA levels, and ameliorated EAE severity [66]. Combined treatment with the synthetic NA precursor L-DOPS and the reuptake inhibitor atomoxetine produced a substantially greater increase in central NA levels and a more pronounced reduction in disease severity than either agent alone, underscoring the importance of achieving sufficient central NAergic augmentation rather than simply blocking reuptake [6]. More recently, chemogenetic activation of LC NAergic neurons reduced demyelination and glial activation in the spinal cord and prefrontal cortex, with the greatest benefit when activation was initiated early in the disease course [67]. Taken together, these studies support the LC as an active regulator of CNS inflammation and tissue integrity, in which NAergic failure exacerbates demyelinating pathology, whereas restoration of LC function confers neuroprotection.

**Fig. 1:**
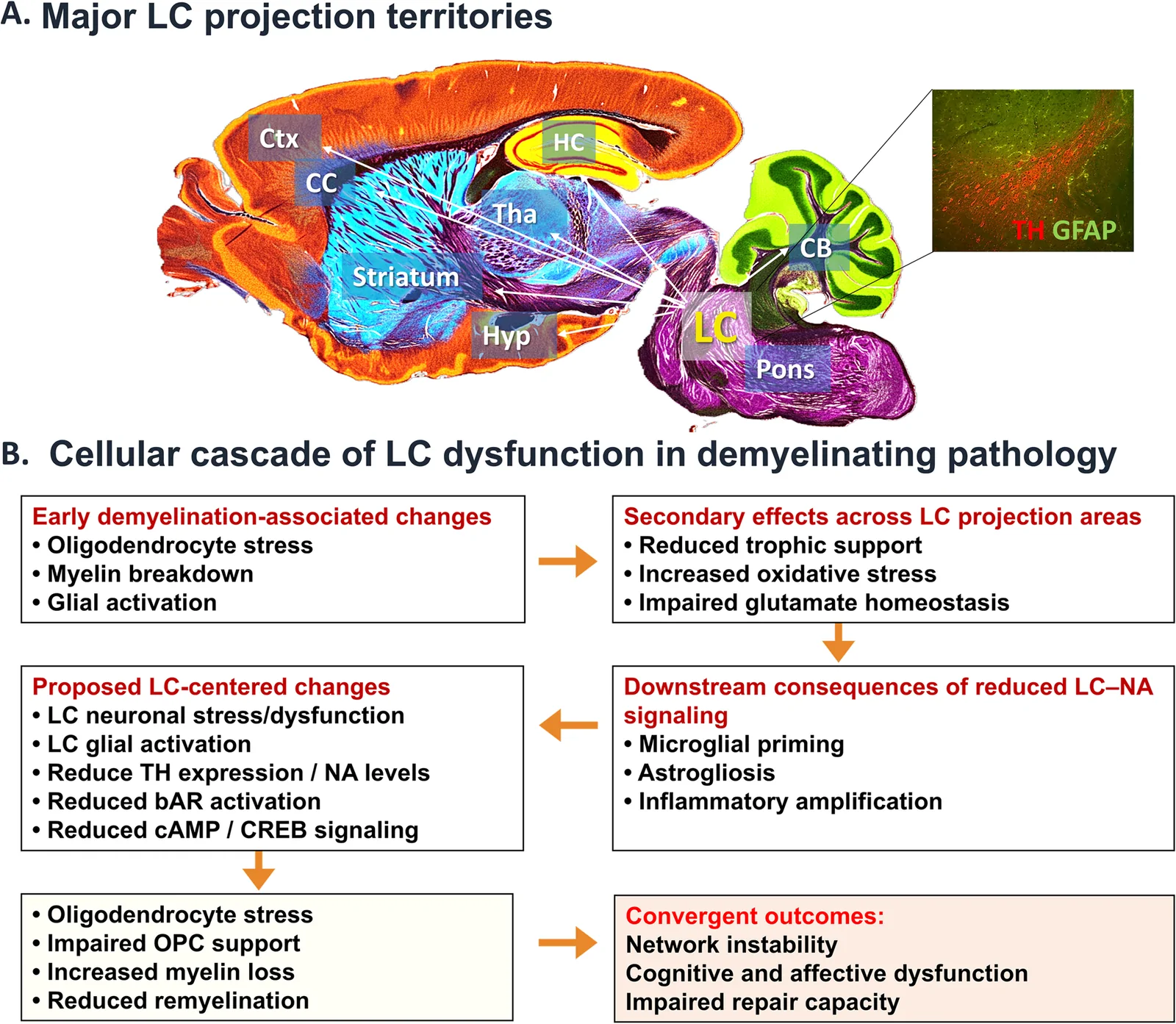
Proposed working model of the locus coeruleus (LC)-centered mechanistic cascade and relevance to Multiple Sclerosis. **A** Major LC projection territories. Sagittal schematic of the mouse brain showing the locus coeruleus (LC), which lies in the pons, and its principal projection targets, including the cortex (Ctx), hippocampus (HC), thalamus (Tha), hypothalamus (Hyp), and cerebellum (CB); corpus callosum (CC) and striatum. The inset shows double immunofluorescence of the LC region for tyrosine hydroxylase (TH, red), which labels noradrenergic neurons, and glial fibrillary acidic protein (GFAP, green), which labels astrocytes. **B** Cellular cascade of LC dysfunction in demyelinating pathology. A scheme to summarize experimentally observed and hypothesized interactions contributing to LC dysfunction in demyelinating pathology; it is provided as a working model for hypothesis generation. Early demyelination-associated changes (oligodendrocyte stress, myelin breakdown, and glial activation) are followed by secondary effects across LC projection regions (reduced trophic support, increased oxidative stress, and impaired glutamate homeostasis) and by downstream consequences of reduced LC-NA signaling (microglial priming, astrogliosis, and inflammatory amplification). These processes converge on proposed LC-centered changes, comprising LC neuronal stress and glial activation with reduced tyrosine hydroxylase (TH) expression and noradrenaline (NA) availability. The accompanying reduction in beta-adrenergic receptor (β-AR) and cAMP/CREB signaling releases the anti-inflammatory brake, and reduces trophic support normally provided by NA, which, together with elevated cytokines, promotes further oligodendrocyte stress, impaired oligodendrocyte precursor cell (OPC) support, reduced remyelination, and increased myelin breakdown, forming a self-amplifying loop. The convergent outcomes of the cascade are network instability, cognitive and affective dysfunction, and impaired repair capacity. *Abbreviations*: CREB, cAMP responsive element binding protein; NA, noradrenaline; OPC, oligodendrocyte progenitor cell; TH, tyrosine hydroxylase

These preclinical findings can be integrated into a working model of LC-centered vulnerability in demyelinating disease (Fig. 1). Primary OLG stress, myelin breakdown, and glial activation may converge with LC neuronal stress and reduced TH-associated NAergic tone. Impaired LC-NA signaling in turn amplifies inflammatory signaling, oxidative stress, and deficient remyelination support across distributed projection territories, contributing to broader network instability and neuropsychiatric dysfunction.

### Clinical and biochemical evidence of LC-NA dysfunction

Impaired LC-NA function is expected to blunt downstream cAMP/PKA/CREB signaling and the CREB-dependent transcription that supports neurotrophin expression, repair programs, and anti-inflammatory restraint, implicating that LC injury in MS has consequences beyond monoamine deficiency alone. Clinical studies support LC-NA dysregulation, although no single biochemical marker yet serves as a definitive surrogate of LC injury. Early cerebrospinal fluid studies found altered NA and metabolite levels; methoxy-hydroxyphenylglycol concentrations were inversely related to disease duration and relapse burden [68, 69]. A multimodal strategy combining catecholamine metabolites, neuromelanin-sensitive LC imaging, spectroscopy, and tract-based structural or functional measures may therefore offer a more informative readout of LC integrity.

More recently, biochemical and molecular analyses have identified markers altered by LC stress and injury. In mice exposed to acute stress to increase LC release of NA, RNA-seq analysis identified a reproducible set of genes in the hippocampus (HC) altered by LC activation, many related to pathways involved in astrocyte energy metabolism [70]. Similarly, RNA-seq identified a large number of genes altered in the LC, but not the nucleus accumbens, following a prolonged stress model of PTSD; many of these were related to morphological changes, growth factors, brain development, and memory [71]. Whether any of these alterations cause associated systemic changes in gene expression and thus provide a biomarker for LC stress remains to be determined.

### Monoaminergic tracts, connectivity, and fatigue

In addition to LC cell body damage, monoaminergic projection pathways are also affected in MS. Fixel-based analysis has shown reduced fiber density and cross-section within dopaminergic mesolimbic pathways, NAergic LC-to-prefrontal tracts, and serotonergic raphe-to-cerebellar projections in people with MS compared to healthy controls [9]. Axonal injury within mesocorticolimbic and LC-to-prefrontal tracts was associated with the severity of cognitive fatigue, supporting the notion that disruption of monoaminergic tracts contributes to central fatigue [10]. In a complementary resting-state fMRI study, reduced functional connectivity was observed between the VTA and default-mode network hubs, between the LC and right prefrontal cortex, and between raphe nuclei and the cerebellum in people with MS [9]. This pattern aligns with the known roles of these circuits in executive control, motivation, and affect, and matches the cognitive and mood symptoms commonly experienced in MS.

It has been reported that people with MS without self-reported cognitive fatigue show increased axial and radial diffusivity in fibers connecting the right posterior hypothalamus and the right LC, while those with fatigue do not exhibit this change and do not differ in lesion load or global atrophy. This pattern has been interpreted as a possible compensatory adaptation helping to maintain arousal by preventing excessive downregulation of wakefulness-related networks [12]. Reduced N-acetylaspartate/creatine (NAA/Cr) ratios in the right pontine LC region also predict selective auditory attention deficits in early RRMS, indicating that LC integrity has measurable functional consequences even when EDSS scores are low [11].

### Infratentorial lesions and mood

Neuropsychiatric symptoms are common in MS, but their anatomical foundations are still not well understood. In a recent infratentorial lesion-mapping study, depression and anxiety were examined in relation to lesions in the raphe nuclei, the LC, and cerebellar lobule VIIA [72]. Depressive symptoms were significantly linked to lesions in both the raphe nuclei and the LC, while anxiety showed a possible trend-level association with LC lesions that did not reach standard statistical significance. These findings provide anatomical evidence that structural damage to brainstem monoaminergic nuclei increases vulnerability to mood disorders in MS, especially depression. Together, the evidence from neuropathology, molecular biology, toxicology, and structural and functional neuroimaging supports a multimodal model of LC-NA involvement in MS (Table 1).

**Table 1:** Multimodal evidence supporting involvement of the locus coeruleus-noradrenergic axis in multiple sclerosis

Domain	Specific Mechanism or Observation	Key Findings & Anatomical Targets	Clinical Relevance	Ref
Neuropathology	Examination of MS autopsy tissue and EAE brains for LC pathology.	Morphological alterations of TH + LC neurons, including hypertrophy and shrinkage. LC neuronal damage and gliosis occur independently of local white-matter plaque burden. Increased astrocytosis around LC neurons.	Suggests LC injury as an independent contributor to MS neuropathology, not fully captured by plaque-based staging.	[8]
Molecular Biology	NA activates β-ARs to suppress glial inflammatory activation.	β-AR activation stimulates cAMP/CREB signaling. Suppression of pro-inflammatory cytokine production. Enhanced expression of neurotrophic factors.	Identifies a druggable anti-inflammatory and neurotrophic pathway relevant to glial activation and repair in MS.	[1–3, 47, 48, 55]
Toxicology	LC neurons selectively accumulate heavy metals (‘heavy metal sink’ hypothesis).	Selective accumulation of mercury, aluminum, and silver in LC neurons. Metals may increase LC neuronal vulnerability and potentially contribute to BBB dysfunction.	Raises the possibility of environmental metal exposure as a modifiable risk factor for LC-related pathology; causal evidence in MS remains lacking.	[13, 14, 73, 74]
Neuroimaging (Structural)	Tractography and DTI assessment of monoaminergic projection integrity.	Reduced fiber density in the LC-prefrontal tracts correlates with cognitive fatigue. Reduced integrity in raphe-cerebellar serotonergic tracts. Increased diffusivity within hypothalamic-LC pathways (proposed compensatory adaptation).	Cognitive fatigue: candidate imaging marker for monoaminergic network disconnection.	[10, 12]
Neuroimaging (Functional/Metabolic)	rs-fMRI and spectroscopy assessment of network dynamics and metabolic health.	Reduced functional connectivity between the brainstem and default mode/executive control networks. Reduced NAA/Cr ratios within the pons/LC region correlate with attentional deficits. Depressive and anxiety symptoms are associated with lesions involving the LC and raphe nuclei.	Attentional deficits, depression, and anxiety.	[9, 11, 72]
Neurodegeneration (cross-disease context)	Direct quantification of LC neuronal loss specific to MS post-mortem tissue remains limited.	Cross-disease evidence from Alzheimer’s disease, Parkinson’s disease, Down syndrome, and multiple system atrophy indicates that LC neuronal vulnerability is a recurring feature of neurodegeneration (see main text, Sect. "LC vulnerability across neurodegenerative diseases"	Supports LC failure as a candidate marker of neurodegenerative burden, particularly relevant to progressive MS.	[5, 25, 75–77]

Legend: Evidence categories summarize neuropathological, molecular, toxicological, structural imaging, functional/metabolic imaging, and cross-disease observations relevant to the LC-NA axis in MS. References are numbered according to the manuscript reference list

*BBB* blood-brain barrier, *CREB* cAMP response element-binding protein, *EAE* experimental autoimmune encephalomyelitis, *LC* locus coeruleus, *MS* multiple sclerosis, *NA* noradrenaline, *NAA/Cr* N-acetylaspartate/creatine, *rs-fMRI* resting-state functional MRI, *TH* tyrosine hydroxylase

## Causes and contributors to LC damage

### Loss of trophic support

A plausible contributor to LC vulnerability in MS is loss of trophic support from projection fields. LC neurons maintain extensive axonal arbors and are therefore highly dependent on intact target interactions and retrograde or trans-synaptic trophic signaling (Fig. 1). In MS, widespread cortical, hippocampal, thalamic, and cerebellar pathology could secondarily destabilize LC neurons by diminishing trophic reciprocity across distributed projection networks. This possibility remains underexplored, but it provides a biologically coherent explanation for LC injury that cannot be reduced to local pontine lesions alone. In parallel, impaired cAMP/CREB-dependent transcription may reduce BDNF-linked trophic maintenance, further compromising neuronal repair and myelin support [1, 48].

### BBB loss and neurovascular unit integrity

A second contributor to LC damage is impaired BBB and neurovascular unit integrity. The LC-NA system participates in regulating the neurovascular unit and can influence BBB permeability, endothelial signaling, glial responses, and local blood flow [78]. More broadly, BBB integrity depends on tightly regulated astrocyte-endothelial interactions and neuronal support [79]. In MS, barrier dysfunction may increase LC exposure to circulating inflammatory mediators and toxic molecules; conversely, reduced LC output may weaken one of the endogenous systems that normally help stabilize the inflammatory tone of the neurovascular environment. This reciprocal relationship makes LC dysfunction both a potential consequence and an amplifier of BBB pathology. Since adrenergic pathways support BBB integrity, reduced NAergic signaling from a damaged LC could increase vascular permeability and thereby increase exposure of LC neurons and adjacent regions to circulating toxicants, including heavy metals proposed to accumulate in vulnerable brainstem nuclei [73, 80]. Extracellular vesicles (EVs) represent an additional mechanism of peripheral-central communication. Circulating EVs may interact with endothelial cells and modulate BBB function, and altered EV cargo, including miRNA and protein content modified by systemic toxicant exposure, may reflect or contribute to neuroinflammatory signaling within the CNS [81]. Such mechanisms may represent an additional route by which environmental exposures contribute to glial activation, myelin injury, and disruption of neuroimmune regulation, processes that are normally modulated by the LC-NA system.

### Toxic metal accumulation in LC neurons

The LC has high metabolic activity, dense vascular contacts, and neuromelanin-containing neurons with metal-binding capacity, making it a plausible target for circulating toxicants. Together with other associative findings, this has led to the hypothesis that environmental toxic metals are selectively absorbed by LC neurons, particularly during stress-related increases in firing and blood flow, and that metal-induced injury reduces central NA levels and increases vulnerability to neurodegenerative, demyelinating, and psychiatric disorders [13]. This possibility is supported by post-mortem studies that provide direct evidence of metal accumulation in LC neurons. One of the first reports came from histochemical staining using silver autometallography (AMG) which stains sulphide and selenide derivatives of mercury, silver, and bismuth. In brain sections from a man continuously exposed to mercury for 5 months, AMG revealed mercury in cortico-motor neurons, LC neurons, as well as undefined glial cells [82]. A subsequent study using sections from the same subject combined AMG with immunohistochemistry confirmed selective mercury deposition in cortical astrocytes, grey matter OLGs, cortico-motoneurons, and a subset of LC neurons [83]. The mercury deposits in cortico-motor neurons prompted comparison of the LC between patients with motor neuron disease (MND) and controls [84], and showed that the LC of 88% of MND patients had at least one neuron with heavy metal staining compared to 42% of controls. Of interest was the presence of heavy metal staining in the LC of the one MS patient within the control group, but not in the LC of 3 PD patients. A subsequent larger case-control study of 21 patients with MS and 109 controls using AMG and laser ablation-based elemental bio-imaging detected inorganic mercury, silver, or bismuth in LC neurons in both groups; however, in a subset of MS brains these metals also appeared in widespread blood vessels, OLGs, astrocytes, and neurons; and combinations of iron, silver, lead, aluminum, mercury, nickel, and bismuth were more frequently detected in the LC of MS cases than in controls [14]. Synchrotron X-ray fluorescence microscopy further revealed marked cell-to-cell variation in concentrations of mercury, selenium, iron, copper, and lead within individual LC neurons in brains of 7 women patients with MS, whereas neighboring anterior pons neurons outside the LC contained no mercury and lower copper levels [74]. Experimental studies further demonstrate that heavy metals can directly damage LC neurons: cellular iron concentrations directly affect NA transporter expression in the LC, with protein levels correlated with regional iron concentrations [85]; iron infusion into the rat LC induces lipid peroxidation, NA depletion, TH-positive neuron loss, and induction of apoptosis [86]. The observed uneven elemental burden suggests a possible explanation for heterogeneous LC neuronal loss which could contribute to clinical heterogeneity. At a broader conceptual level, the toxic metal hypothesis proposes that metal-mediated LC damage weakens the BBB in projection territories, allowing circulating toxicants to enter astrocytes and transfer to OLGs and neurons, thereby promoting demyelination and neurodegeneration across MS, amyotrophic lateral sclerosis, PD, and AD [73]. However, experimental evidence directly supporting these hypotheses is still lacking.

### Implications for MS pathogenesis

The implications for MS pathogenesis can be assessed by distinguishing three levels of evidence. Established findings include the direct demonstration by AMG, laser ablation elemental bioimaging, and synchrotron X-ray fluorescence that redox-active metals accumulate in a subset of LC neurons and vary from cell to cell [74, 87]. Associative observations include the more frequent detection of metal combinations in the LC of MS cases than controls in small post-mortem series [14]. Hypothetical mechanisms include the proposal that this accumulation causally drives LC injury, NA depletion, and downstream demyelination; this causal step has not been demonstrated and should be regarded as provisional pending prospective, exposure-controlled studies. Iron has a particularly high affinity for neuromelanin and is detected in the LC in Alzheimer’s disease, aging, Parkinson’s disease, and Huntington’s disease [88, 89]. Mercury has also been reported in the LC of Parkinson’s disease patients [90], whereas is was not detected in the LC of autistic patients [91]. Mercury and related metals can induce oxidative stress, mitochondrial dysfunction, DNA damage, and immune dysregulation in CNS cells, processes that are widely implicated in the pathophysiology of MS [73, 86, 88, 89]. Although causal evidence remains lacking, the toxic metal hypothesis provides a mechanistic framework linking environmental metal exposure, selective LC vulnerability, and downstream demyelination. Reduced NAergic output from a damaged LC could weaken glial anti-inflammatory restraint and barrier stability and may also impair OLG maturation programs that support remyelination. Although epidemiological evidence linking metal exposures to MS risk remains limited, the hypothesis has practical implications, since reductions in environmental metal pollution and occupational exposure could help lessen the burden of LC-linked neurological disorders.

### LC vulnerability across neurodegenerative diseases

LC degeneration is not unique to MS, supporting the idea that this nucleus has inherent and possibly shared biological vulnerabilities. Parallel evidence from AD, PD, Down syndrome, and multiple system atrophy indicates that LC vulnerability is a recurring feature of neurodegenerative disorders, often preceding or amplifying cortical and subcortical pathology. Quantitative histological studies in both humans and rodents showed that LC neuron numbers remain relatively stable during healthy aging [27, 30, 33]. Significant loss of LC neurons projecting to the cerebral cortex was established as a core feature of senile dementia decades ago [25]. Advanced in vivo neuromelanin imaging has directly linked LC structural damage to cognitive dysfunction and altered brain metabolism in multiple system atrophy [75]. This cross-disease convergence reinforces the view that LC failure can magnify neuroinflammation, trophic insufficiency, and network dysfunction even when the primary initiating pathology differs [5, 76, 77]. However, the triggers of this vulnerability differ across disorders. In AD the LC accumulates hyperphosphorylated (pre-tangle) tau decades before cortical pathology and shows severe neuronal loss that correlates with plaque and tangle burden and disease duration, with reductions in central NA. In PD and MSA, alpha-synuclein pathology and loss of melanized neurons affect the LC alongside the SN, and LC neuromelanin loss tracks cognitive impairment and altered brain metabolism [46, 75]. In progressive supranuclear palsy, tau aggregation and NAergic cell loss in the LC relate to clinical severity, and Down syndrome shows early LC tau pathology consistent with its Alzheimer-type trajectory [5]. These shared elements across diverse disorders suggest intrinsic features leading to LC vulnerability: long, highly arborised, metabolically demanding axons; pacemaker activity with a high oxidative load; neuromelanin with metal-binding capacity; and dense vascular contact. The disease-specific elements are the proteinopathies or primary insults that engage these vulnerabilities, namely tau, alpha-synuclein, or, in MS, autoimmune inflammation. Framing MS within this spectrum places LC failure as a convergent amplifier of neuroinflammation and trophic insufficiency rather than a disease-specific lesion, so that loss of NAergic anti-inflammatory restraint may be a common and broadly targetable downstream consequence.

### Sex differences in LC vulnerability

The LC is a sexually dimorphic nucleus, which may be relevant to the female predominance of MS. Preclinical work shows structural and molecular sex differences in LC neurons, including differences in cell number, larger and more complex dendritic arbors in females, greater intrinsic excitability, and sex-biased gene expression ([92, 93]). Female LC neurons are more sensitive to the stress peptide corticotropin-releasing factor and show estrogen-dependent enhancement of NA synthesis, features linked to the higher female prevalence of stress-related and affective disorders [94–96]. In vivo neuromelanin-sensitive MRI likewise reports higher LC signal in women than in men [97]. Sex-dependent differences have also been reported that are dependent upon LC projection areas [98], and led to the proposal that changes in estrogen levels could render the LC more susceptible to damage, which could contribute to increased prevalence of AD in women [99]. Whether these differences modify LC susceptibility to inflammatory or metal-related injury in MS is unknown, but they argue for treating sex as a biological variable in LC-focused MS studies.

## LC, NA, and neuropsychiatric symptoms in MS

### Fatigue, mood, and anxiety symptoms

Fatigue in MS is a heterogeneous and multidimensional symptom that likely reflects interactions between primary disease mechanisms and secondary clinical contributors. Evidence supports associations with immune activation, including pro-inflammatory cytokine signaling such as TNF-α and related inflammatory mediators [100, 101], sleep disturbance [102], depressive symptoms [103], autonomic dysregulation [104], and neuroendocrine/HPA-axis imbalance [105]. Fatigue may also be worsened by medication effects or treatment-related adverse effects [106]. At the neural systems level, MS fatigue has been linked to structural and functional disruption of distributed cortico-thalamo-striatal, cortico-limbic, and monoaminergic networks, rather than to a single anatomical lesion or pathway [107]. Within this framework, LC–NA dysfunction should be viewed as one mechanistically plausible contributor, not as the sole cause of MS fatigue. Its relevance lies in the fact that the LC–NA system can influence arousal, attention, cognitive effort, autonomic regulation, and neuroimmune signaling, which is likely to vary across individuals.

The circuit- and cellular-level abnormalities described above provide a plausible framework for understanding fatigue and affective symptoms in MS. As detailed in Sect. "Monoaminergic tracts, connectivity, and fatigue", fixel-based imaging and tractography studies have identified injury within NAergic LC–prefrontal and hypothalamic–brainstem pathways, with greater tract disruption associated with more severe cognitive fatigue. These findings support a network-level substrate for fatigability rather than a purely lesion-count-based explanation [9, 10, 12]. Mood symptoms may overlap mechanistically with fatigue rather than representing an entirely separate domain. As described in Sect. "Infratentorial lesions and mood", infratentorial lesion-mapping studies have linked depressive symptoms to structural damage involving the LC and raphe nuclei [72], implicating disruption of NAergic and serotonergic systems. In parallel, clinical evidence that serotonin–noradrenaline (NA) reuptake inhibitors, such as duloxetine, can improve depressive symptoms in MS cohorts supports the therapeutic relevance of monoaminergic pathways, although this does not establish LC dysfunction as the sole mechanism [108]. Together, these data support a shared monoaminergic and network-level framework through which fatigue, mood disturbance, and anxiety-related symptoms may emerge in MS.

### Cognitive dysfunction and monoaminergic modulation

Cognitive impairment affects many patients with MS and commonly involves slowed processing speed, executive dysfunction, episodic memory problems, and impaired learning. A recent network meta-analysis suggested low-certainty benefits of stimulant, glutamatergic, and NAergic agents on selected cognitive tests, with L-amphetamine showing possible improvement in memory function, but the evidence remained fragmented, and head-to-head comparisons were limited [109]. Atomoxetine, a selective NA reuptake inhibitor, has shown preliminary domain-specific cognitive benefits in clinical studies; these are discussed in detail in Sect. "NA reuptake inhibitors, SNRIs, and combination approaches" [110].

## β₂-agonists, NA reuptake inhibitors, and LC-targeted therapies

### Preclinical evidence: LC in the EAE model

As reviewed in Sect. "Preclinical biochemical evidence of LC-NA dysfunction", experimental NA depletion worsens inflammation and tissue injury in EAE, while NA-augmenting strategies, including vindeburnol, catalpol, combined L-DOPS/atomoxetine, and chemogenetic LC activation, reduce disease severity, demyelination, and glial activation. This causal evidence provides the experimental basis for LC-NA-directed clinical strategies in MS.

### β_2_-adrenergic agonists in clinical studies

β_2_-adrenergic agonists such as albuterol (salbutamol) increase intracellular cAMP and reduce interleukin-12 (IL-12) production in monocytes and dendritic cells, shifting T helper 1 cell responses away from pro-inflammatory phenotypes [111–113]. In early open-label work in secondary progressive MS, short-term oral salbutamol was associated with reduced IL-12 production by monocytes and dendritic cells, with suppression persisting for approximately one week after drug discontinuation [113]. In a key randomized controlled trial (ClinicalTrials.gov: NCT00039988), patients with RRMS receiving glatiramer acetate plus albuterol showed improved Multiple Sclerosis Functional Composite scores at six and twelve months, a delayed time to first relapse, and immunological changes consistent with reduced production of IL-13 and interferon-gamma (IFN-γ) [111]. On this basis, a narrative review concluded that salbutamol may represent a promising add-on immunomodulatory therapy in MS, while emphasizing the need for larger definitive trials [112]. Population-based pharmaco-epidemiological studies provide additional support. In a nationwide Taiwanese case-control study, β2-adrenergic agonist use was linked to a dose-dependent decrease in MS risk, with adjusted odds ratios below one across different exposure levels [114]. In a separate registry-based study, individuals later diagnosed with MS had fewer prescriptions for ipratropium or salmeterol than matched controls, and modeling indicated that use of these medications was associated with a lower chance of an eventual MS diagnosis [115]. However, the therapeutic effects of β₂-adrenergic agonists are not uniform across clinical outcomes. In a pilot randomized placebo-controlled trial in RRMS, oral salbutamol did not show superiority over placebo on fatigue outcomes, and no clinical benefit on disability measures was detected [116]. These neutral findings highlight that domain-specific outcomes, dosing schedules, CNS penetration, and patient heterogeneity could influence response.

### NA reuptake inhibitors, SNRIs, and combination approaches

In patients with MS, atomoxetine has shown encouraging but preliminary effects on cognition. In a small randomized placebo-controlled trial, three months of atomoxetine treatment were associated with improvements in processing speed and performance on verbal learning and visual memory tests from the MACFIMS battery [110], whereas improvements in attention and cognitive speed were reported without corresponding gains in memory measures, indicating domain-specific and time-limited effects [117]. Clinical work with duloxetine in MS has shown benefit for depressive symptoms and fatigue [108], and a separate pilot study suggested a benefit for overactive bladder symptoms [118]. Venlafaxine, a serotonin-NA reuptake inhibitor (SNRI), has been shown in EAE to ameliorate disease severity through suppression of pro-inflammatory cytokines, including IL-12 p40, TNF-alpha, and IFN-gamma in encephalitogenic T cells, splenocytes, and macrophages, while simultaneously increasing BDNF expression in inflamed CNS tissue [119]. This convergent neuroimmune and neurotrophic profile suggests that SNRIs may exert benefits on both mood-related and immunological pathways.

Combination approaches have also been explored. In a randomized placebo-controlled trial of the Cari Loder regimen, all participants received weekly intramuscular vitamin B12, and the addition of oral lofepramine and L-phenylalanine produced a small but statistically significant additional improvement in disability and symptom scores, with changes becoming evident within a few weeks [120]. Activation of NAergic systems in the LC and lateral tegmentum was proposed as a possible mechanism, although this has not been directly tested [120, 121]. A summary of preclinical and clinical interventions targeting the LC-NA axis is provided in Table 2.

**Table 2:** Preclinical and clinical interventions targeting the locus coeruleus-noradrenergic axis in multiple sclerosis

Intervention / Agent	Study Type	Proposed Mechanism of Action	Key Outcomes / Findings	Primary Therapeutic Target	Ref
β2-Adrenergic Agonists					
Salbutamol (Albuterol)	Clinical (randomized controlled trial)	Increases cAMP signaling; suppresses Th1 immune responses via reduced IL-12 production.	Improved MS Functional Composite scores at 6 and 12 months but not sustained at 24 months; delayed time to first relapse; changes in the primary immunologic endpoints (IL-13 and IFN-gamma).	Immunomodulatory	[111–113]
Salbutamol (Albuterol)	Clinical (pilot randomized controlled trial)	Immunomodulation and potential monoaminergic stimulation.	Negative result: no significant advantage over placebo for fatigue severity (FSS) or neurological impact (NFI-MS) at 90 days.	Immunomodulatory	[116]
Fenoterol	Clinical (case-control study)	β2-adrenergic agonism; increases cAMP signaling.	Associated with a dose-dependent reduction in MS risk (adjusted odds ratios below 1).	Immunomodulatory	[114]
Salmeterol / Ipratropium	Clinical (case-control study)	β2-adrenergic agonism (salmeterol).	Associated with a reduced likelihood of subsequent MS diagnosis.	Immunomodulatory	[115]
Reuptake Inhibitors (NRIs & SNRIs)					
Atomoxetine	Clinical (randomized controlled trial; corroborated by network meta-analysis)	Selective noradrenaline reuptake inhibitor; modulates frontoparietal networks.	Significant improvements in processing speed and memory (CVLT, BVMT-R, SDMT) over 3 months.	Neuromodulatory / cognitive	[109, 110]
Atomoxetine	Clinical (conference abstract)	Selective noradrenaline reuptake inhibitor.	Short-term improvement in processing speed and verbal learning; no durable effect on memory. A distinct cohort from the RCT above.	Neuromodulatory / cognitive	[117]
Duloxetine	Clinical (open-label study)	Serotonin-noradrenaline reuptake inhibitor; dual action on mood and descending pain/autonomic pathways.	Significant reduction in depression (BDI) and fatigue (MFIS); improved overactive bladder symptoms and quality of life.	Neuromodulatory / symptomatic	[108, 118]
Venlafaxine	Preclinical (EAE model)	Serotonin-noradrenaline reuptake inhibitor; suppresses pro-inflammatory cytokines (IL-12 p40, TNF-alpha, IFN-gamma).	Ameliorated EAE course; increased CNS BDNF expression.	Neuroprotective/immunomodulatory	[119]
Precursors & LC-Targeted Agents					
Atomoxetine + L-DOPS	Preclinical (EAE model)	A combination of a noradrenaline reuptake inhibitor and a synthetic noradrenaline precursor.	Improved clinical scores and dampened astrogliosis; superior to either agent alone; no effect on peripheral Th1/Th17 cytokine production.	Neuroprotective/astroglial modulation	[6]
Vindeburnol	Preclinical (EAE model)	Vincamine derivative; enhances tyrosine hydroxylase expression and LC neuron survival.	Improved clinical scores; reduced cerebellar demyelination; normalized spinal cord NA levels when given after onset.	Neuroprotective	[7]
Catalpol	Preclinical (EAE model)	Increases LC noradrenaline output.	Raised spinal cord NA levels; ameliorated EAE severity.	Neuroprotective	[66]
Lofepramine + L-Phenylalanine + B12	Clinical (randomized controlled trial)	Tricyclic antidepressant plus amino acid precursor and cofactor; hypothesized to activate LC and lateral tegmental systems.	Improved symptoms across MS subtypes with rapid onset of effect.	Neuromodulatory / symptomatic	[120, 121]
L-Amphetamine	Clinical (network meta-analysis)	CNS stimulant; enhances monoaminergic neurotransmission.	Signals suggesting potential improvement in memory domains; evidence is fragmented.	Neuromodulatory / cognitive	[109]

Legend: Preclinical and clinical interventions are grouped by pharmacological or mechanistic category. The table summarizes reported mechanisms and outcomes but does not imply proven efficacy across all MS phenotypes. References are numbered according to the manuscript reference list

*BDNF* brain-derived neurotrophic factor, *cAMP* cyclic adenosine monophosphate,* CNS* central nervous system, *EAE* experimental autoimmune encephalomyelitis,* IFN-gamma* interferon-gamma,* IL-12* interleukin-12, *LC *locus coeruleus, *L-DOPS* L-threo-3,4-dihydroxyphenylserine, *MS* multiple sclerosis, *NA* noradrenaline, *NFI-MS* Neurological Fatigue Index for MS, *SNRI* serotonin-noradrenaline reuptake inhibitor, T*NF-alpha* tumor necrosis factor-alpha

### Limitations of the current therapeutic evidence

Despite the mechanistic appeal of LC-NA-targeted interventions, the clinical evidence base in MS remains limited by small sample sizes, short treatment durations, heterogeneous outcome measures, and minimal stratification by markers of LC or monoaminergic dysfunction. As illustrated by the neutral salbutamol fatigue trial (Sect. "β2-adrenergic agonists in clinical studies") and the domain-limited atomoxetine findings, reported benefits have generally been modest and domain-specific, and caution against direct translation of preclinical findings into routine practice. Moreover, very few studies have incorporated LC imaging, quantitative assessment of monoaminergic tract integrity, or characterization of environmental exposures into their inclusion criteria or stratification strategies. As a result, patient subgroups who might be particularly responsive, such as those with demonstrable LC injury, high cognitive fatigue burden, or prominent depressive symptoms, may have been diluted within unselected trial cohorts.

## Systems model: the LC as a hub integrating toxicants, neuroinflammation, and symptoms

**Fig. 2:**
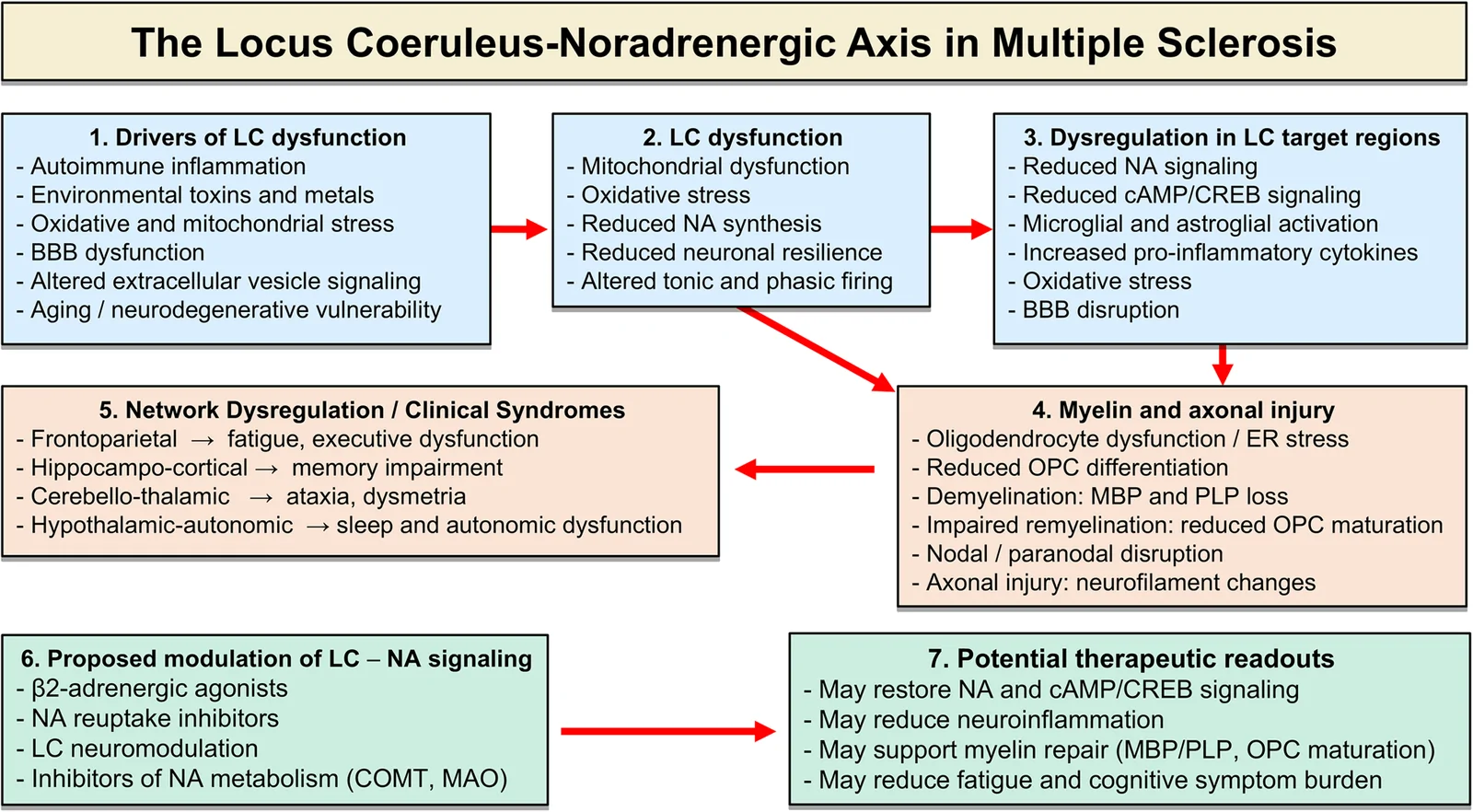
Multi-level model of locus coeruleus–noradrenergic axis dysfunction in multiple sclerosis. Schematic representation of a proposed multi-level framework linking upstream drivers of locus coeruleus–noradrenergic (LC–NA) dysfunction to downstream cellular and tissue-level injury, network-level dysregulation, clinical consequences, and possible therapeutic approaches in multiple sclerosis (MS). Multiple drivers (box 1) converge to induce LC dysfunction (box 2) culminating in reduced NA output, leading to dysregulation in LC target regions (box 3). These changes, which include glial cell activation, reduced trophic support, oxidative stress and loss of BBB integrity can contribute to oligodendrocyte and axonal injury, myelin loss, and alterations in nodal and paranodal organization (box 4). In addition, these changes could impair limited attempts at remyelination. We propose that the consequences of these combined perturbations interact with ongoing network pathologies (box 5) exacerbating behavioral syndromes including fatigue, memory impairment, ataxia, and other autonomic functions. To address this, we propose interventions to modulate the LC-NA axis (box 6) thereby increasing NA production or availability, reducing NA breakdown, activating b2-AR signaling, or potentially rescuing damaged LC neurons; which together are expected to result in beneficial outcomes (box 7) that limit further damage or potentially restore target area homeostasis. The interactions we illustrate in this schematic are derived from partially supported mechanisms, or proposed mechanistic links based on the correlative findings discussed. The model focuses on LC dysfunction, however it exists within the context of broader monoaminergic pathology. Abbreviations: BBB, blood–brain barrier; cAMP, cyclic adenosine monophosphate; CREB, cAMP response element-binding protein; ER, endoplasmic reticulum; LC, locus coeruleus; MBP, myelin basic protein; MS, multiple sclerosis; NA, noradrenaline; OPC, oligodendrocyte precursor cell; PLP, proteolipid protein

Taken together, the LC can be viewed as a systems-level hub in MS (Fig. 2), characterized by the following:

1. Upstream vulnerability. Environmental exposures, especially toxic metals, may accumulate selectively in LC neurons, where variable elemental burden and morphological signs of stress have been observed in autopsy material and MS brains [13, 14, 83, 122]. Genetic susceptibility and chronic disruption of NA synthesis may further increase vulnerability.
2. Loss of intrinsic anti-inflammatory control. Reduced LC output weakens a major endogenous mechanism that normally restrains microglial and astrocytic activation, cytokine production, and oxidative stress through β-adrenergic signaling. Under such conditions, immune triggers are more likely to elicit exaggerated or poorly resolved glial activation and microglial priming [1–3].
3. Network-level consequences. Damage to the LC and related monoaminergic tracts is associated with altered connectivity and with fatigue, attentional deficits, and depressive symptoms in MS, supporting a role for monoaminergic network disconnection in symptom expression [9–12, 72].
4. Interaction with demyelination and neurodegeneration. Impaired LC signaling may permit more aggressive or poorly resolved inflammatory injury, whereas restoration of NAergic tone improves demyelination, glial activation, and neuroprotective signaling in EAE [6, 7, 66, 67, 119, 123]. Availability of standardized g-ratio analysis tools could further quantify myelin integrity and connect LC-targeted biology to OLG repair outcomes in preclinical work [123].
5. Therapeutic leverage within a hub-based framework. LC-NA-directed therapies may be most useful as biomarker-guided, add-on strategies aimed at selected symptom domains or patient subgroups, particularly those with demonstrable LC injury, high cognitive fatigue burden, or prominent depressive symptoms. Rather than replacing immunomodulatory therapies, LC-centered modulation may function as a complementary strategy that restores intrinsic regulatory balance and mitigates symptom burden

The myelin and axonal injury module in Fig. 2 is supported by evidence from human MS tissue, experimental demyelination models, MRI–pathology correlation studies, and serum biomarker cohorts. However, the specific directional links between LC–NA dysfunction and these downstream myelin/axon injury pathways remain partly hypothetical and require direct experimental validation.

### The LC within a broader monoaminergic and network context

The LC does not act in isolation, but functions as a hub within a broader monoaminergic network that is disrupted in MS, and must be considered within this context when considering therapeutic leverage. While best characterized for NA synthesis and release, the LC is also a major source of dopamine (DA), the immediate precursor of NA. LC-derived DA is released in the HC [124, 125] and thalamus [126], where it is involved in memory and stress responses. In some cases NA and DA are co-released from the same neurons [127]. In contrast, LC neurons do not express 5-hydroxy-tryptamine (serotonin); instead the LC receives serotonergic afferents originating in the dorsal raphe nucleus [128]. Similarly, while LC NAergic damage is well established, damage to both DAergic and serotonergic neurons occurs in neurodegenerative diseases including AD and PD. In MS, serotonergic raphe and DAergic mesolimbic systems are also affected, and fixel-based imaging shows reduced fiber density across raphe-to-cerebellar and ventral tegmental projections alongside LC-to-prefrontal tracts, with overlapping contributions to fatigue, affect, and cognition [10]. Reduced serotonin and DA availability are also implicated in MS fatigue and mood disturbance [107]. The clinical picture, therefore, reflects distributed monoaminergic and network-level pathology, with LC-NA dysfunction as one component. We emphasize the LC since it is the principal source of CNS NA [129], exerts distinctive anti-inflammatory and trophic influences on glia and OLGs [1, 2], and is selectively vulnerable to toxin accumulation and stress [14, 87, 130].

### LC dysfunction across MS phenotypes

MS phenotypes differ in their dominant biology. Relapsing-remitting MS is characterized by focal, blood-brain barrier-permeable inflammatory white matter lesions, whereas progressive MS is dominated by compartmentalized meningeal and parenchymal inflammation behind a relatively intact barrier, with diffuse grey matter demyelination, neuroaxonal loss, and microglial activation that track disability and cognitive decline more closely than focal plaque burden [131, 132]. The LC is well placed to contribute to this progressive, neurodegenerative phase: its widespread cortical and deep grey matter projections [129], its role in restraining glial activation [51], and its support of neurotrophic and OLG repair programs [67] are all most relevant to the diffuse pathology that defines progression. Consistent with this, experimental NA depletion worsens [6, 8], and NA augmentation reduces demyelination and glial activation independently of acute relapse activity [6, 67]. We therefore propose the testable hypothesis that LC-NA dysfunction is more closely linked to smoldering inflammation and neurodegenerative progression in MS than to acute relapse activity. In this framework, LC pathology may contribute to chronic network inefficiency, fatigue, impaired arousal regulation, and progressive tissue vulnerability. Accordingly, LC-directed strategies may be most relevant as adjunctive neuroprotective or neuromodulatory approaches in progressive MS or in relapsing patients with progression independent of relapse activity. However, direct comparative data on LC pathology across relapsing-remitting and progressive MS remain limited, and stratified neuropathological, neuromelanin-sensitive MRI, tractography, and longitudinal clinical-imaging studies are needed to test this prediction.

## Future directions

Several limitations temper the current conclusions. Much of the neuropathological and toxic metal evidence comes from small post-mortem series biased toward progressive or late-stage disease; similar studies in early-stage MS, including radiologically isolated syndrome (RIS) and clinically isolated syndrome (CIS) could help to determine if toxic exposures play a role in early disease evolution. Longitudinal in vivo LC imaging has not yet been systematically implemented in MS, and toxic metal studies remain observational, limiting causal inference. Most interventional studies of β₂-AR agonists and NAergic modulators have been small, short, and methodologically heterogeneous; standardization across studies will be essential to draw stronger conclusions. For patient stratification, the most realistic near-term readouts are neuromelanin-sensitive MRI of the LC, diffusion-based measures of LC-to-prefrontal and hypothalamic-LC tract integrity, and magnetic resonance spectroscopy (NAA/Cr) in the pontine LC region, complemented, where feasible, by quantitation of catecholamine metabolites. Neuromelanin-sensitive MRI is the most well developed, has been validated histologically [133] and shows good reproducibility and disease sensitivity in PD [134], AD [135], and MSA [136]. This has been facilitated by automated LC segmentation pipelines [137]. although routine clinical application is still limited by technical limitations (i.e. differences in scanners, protocol standardization) and overall usage that could influence LC signal measurements. Despite this, it application to MS remains untested, as have studies of neuromelanin in MS. Neuromelanin-MRI therefore presents a promising but not yet validated MS biomarker. Other candidate LC-related biomarkers associated with clinical manifestations, and potential therapeutic strategies for future biomarker-enriched studies are summarized in Table 3.

**Table 3:** LC-related biomarkers, clinical correlates, and candidate therapeutic strategies in multiple sclerosis

Biomarker / Strategy	Modality	Validation Status in MS	Associated Clinical Manifestations	Candidate Therapeutic Strategy	References
Neuromelanin-sensitive MRI (NM-MRI)	Structural MRI	Validated histologically and has good reproducibility in PD, AD, and MSA. Its use in MS is lacking	LC structural integrity: a candidate surrogate for LC neuronal loss.	Patient stratification for LC-targeted trials.	[133–137]
Diffusion tractography (LC-prefrontal and hypothalamic-LC tracts)	Diffusion MRI fixel-based analysis	Demonstrated in MS cohorts. Not yet standardized for routine clinical use.	Cognitive fatigue; attentional deficits.	Stratification by tract-level LC network integrity.	[9, 10, 12]
Magnetic resonance spectroscopy (NAA/Cr, pontine LC region)	MR spectroscopy	Demonstrated in early relapsing-remitting MS in small cohorts to date.	Selective attention deficits, even at low EDSS.	Early identification of LC-related cognitive risk.	[11]
CSF/plasma catecholamine metabolites (e.g., MHPG)	Biochemical assay	Preliminary and associative. Not validated as a standalone biomarker.	Disease duration and relapse burden.	Adjunct biomarker for monitoring LC-NA tone alongside imaging.	[68, 69]
β2-Adrenergic agonists (e.g., salbutamol)	Pharmacological Immunomodulatory	Mixed clinical trial evidence; positive on relapse-related composite outcomes, neutral on fatigue.	Relapse activity; immune activation.	Add-on immunomodulatory therapy.	[111, 116]
NA reuptake inhibitors / SNRIs (e.g., atomoxetine, duloxetine)	Pharmacological Neuromodulatory	Preliminary, domain-specific clinical benefit in small trials.	Cognitive dysfunction, fatigue, and depression.	Add-on cognitive- or mood-targeted therapy.	[108, 110]

Legend: Candidate biomarkers and therapeutic strategies are intended for hypothesis-generating, biomarker-enriched studies. Validation status refers specifically to MS unless otherwise stated. References are numbered according to the manuscript reference list

*AD* Alzheimer's disease, *Cr* creatine, *LC* locus coeruleus, *MHPG* 3-methoxy-4-hydroxyphenylglycol, *MS* multiple sclerosis, *MSA* multiple system atrophy, *NA* noradrenaline, *NAA* N-acetylaspartate, *NM-MRI* neuromelanin-sensitive MRI,* PD* Parkinson's disease, *SNRI* serotonin-noradrenaline reuptake inhibitor

Whether current disease-modifying therapies indirectly engage LC-related pathways is also untested. While there are promising results from small trials using NA-reuptake inhibitors or dual SNRIs [108, 120, 121], it is not known whether those treatments had any direct effect on NAergic neurons. Similarly, although several FDA-approved drugs enhance NA output from the LC or reduce NA breakdown (droxidopa, atomoxetine, modafinil, albuterol) there is no data supporting protective effects on LC neurons. Vindeburnol, thought to increase LC TH expression, and which is effective in EAE [7], was never approved for clinical use. Future work could therefore prioritize biomarker-enriched clinical trials that stratify participants by LC imaging, monoaminergic tract integrity, and neuropsychiatric phenotype. Incorporating environmental exposure data and genetic susceptibility factors, including those affecting detoxification capacity and mitochondrial resilience, will be important for testing toxicant-related hypotheses. Mechanistically informed combination strategies, such as pairing NA-augmenting agents with disease-modifying therapies, remyelination approaches, or cognitive rehabilitation, can be carried out in pre-clinical models (EAE, cuprizone) models. Finally, cross-disease comparisons between MS, AD, PD, and psychiatric disorders may help distinguish shared versus disease-specific contributions of the LC to pathology. Overall, we suggest that any LC-directed interventions should be viewed as potential adjunctive symptomatic or neuroprotective approaches rather than established disease-modifying therapies, that would complement, rather than replace, treatments targeting peripheral and central immune mechanisms in MS.

### Conflicting findings and unresolved questions

Several lines of evidence complicate a simple LC-centered account. Early in EAE, NA levels can rise before falling, suggesting a dynamic, possibly compensatory response rather than uniform depletion [8]. LC damage correlates only weakly with focal plaque burden, which supports the hub argument but also leaves the relationship between LC injury and conventional lesion measures unresolved [8]. Clinical trials are not uniformly positive: oral salbutamol failed to improve fatigue in a placebo-controlled pilot [116], and the cognitive benefits of NAergic agents are small, domain-specific, and sometimes dissociated from memory outcomes [110]. The toxic metal data derive from small, late-stage post-mortem series and cannot establish causation or temporal order [14, 87]. Neuromelanin MRI is not yet validated in MS. Finally, because serotonergic and dopaminergic systems are co-affected, attributing specific symptoms to the LC alone risks overinterpretation [138]. We therefore present the LC-NA framework as a hypothesis-generating synthesis that is consistent with, but not proven by, current data, and that requires prospective, stratified, and adequately powered testing.

## Conclusions

The LC is more than an incidental casualty of MS. Its unique biological properties place it at the intersection of neuroinflammatory regulation, environmental vulnerability, and network-level symptom expression. Disruption of proper LC-NA output has the potential to remove a critical endogenous brake on glial cell activation, resulting in disruption of monoaminergic circuits supporting attention, mood, and arousal, and compromising necessary OLG repair programs. The convergence of neuropathological, toxicological, imaging, and experimental evidence reviewed here is consistent with LC-NA dysfunction as an active and potentially modifiable contributor to MS progression rather than a secondary epiphenomenon. At the same time, the LC represents one influential node within a broader monoaminergic and network-level pathology, and we advance it as a hub in that qualified sense rather than as a single master regulator. We suggest that a biomarker-guided strategy combining NAergic interventions with indicators of glial activity, myelin integrity, and environmental exposure could help identify patient subgroups most likely to benefit, moving beyond the unselected trial designs that have limited progress to date. Appreciating the LC as a systems-level hub opens a coherent and underexplored therapeutic avenue that complements existing disease-modifying strategies in MS.

## Data Availability

No datasets were generated or analysed during the current study.

## Abbreviations

AD: Alzheimer disease
β-AR: β-adrenergic receptor
BBB: blood-brain barrier
BDNF: Brain-derived neurotrophic factor
cAMP: Cyclic adenosine monophosphate
CIS: Clinically isolated syndrome
CC: Corpus callosum
CNS: Central nervous system
Cr: Creatine
COMT: Catechol-O-methyltransferase
CREB: CAMP response element-binding protein
CRF: Corticotropin-releasing factor
DA: Dopamine
DAergic: Dopaminergic
DBH: Dopamine β-hydroxylase
DMN: Default-mode network
DMT: Disease-modifying therapy
DNA: deoxyribonucleic acid
DOPS: L-threo-dihydroxyphenylserine (droxidopa)
EAE: Experimental autoimmune encephalomyelitis
EDSS: Expanded Disability Status Scale
EV: Extracellular vesicle
GFAP: Glial fibrillary acidic protein
GM: Grey matter
HPA: Hypothalamic-pituitary-adrenal
HC: Hippocampus
IFN-γ: Interferon gamma
IL-12: Interleukin-12
LC: Locus coeruleus
MACFIMS: Minimal Assessment of Cognitive Function in MS
MAO: Monoamine oxidase
MHPG: 3-methoxy-4-hydroxyphenylglycol
miRNA: MicroRNA
MND: Motor neuron disease
MRI: Magnetic resonance imaging
MS: Multiple sclerosis
MSA: Multiple system atrophy
NA: Noradrenaline
NAA: N-acetylaspartate
NAergic: Noradrenergic
NARI: Noradrenaline reuptake inhibitor
NET: Norepinephrine transporter
NF-κB: Nuclear factor kappa B
NGF: Nerve growth factor
NM-MRI: Neuromelanin-sensitive magnetic resonance imaging
Nrf2: Nuclear factor erythroid 2-related factor 2
OLG: Oligodendrocyte
OPC: Oligodendrocyte precursor cell
PD: Parkinson’s disease
PKA: Protein kinase A
PMS: Progressive multiple sclerosis
PPMS: Primary progressive multiple sclerosis
PSP: Progressive supranuclear palsy
PTSD: Post-traumatic stress disorder
RIS: Radiologically isolated syndrome
RNA-seq: RNA sequencing
RRMS: Relapsing-remitting multiple sclerosis
SN: Substantia nigra
SNRI: Serotonin-noradrenaline reuptake inhibitor
SPMS: Secondary progressive multiple sclerosis
TH: Tyrosine hydroxylase
Tha: Thalamus
TNF: Tumor necrosis factor
TUNEL: Terminal deoxynucleotidyl transferase dUTP nick-end labeling
VTA: Tentral tegmental area
WM: White matter
